# NSSI contagion in adolescent friendships: exploring the impact of peer influence

**DOI:** 10.1186/s13034-025-00946-w

**Published:** 2025-08-18

**Authors:** Chunxi Ke, Zhiruo Zhou, Zhixuan Ren, Xiaoshu Li, Hairuo He, Yafei Chen, Mengjun Liu, Yunheng Yao, Yumeng Ju, Yan Zhang

**Affiliations:** 1 Department of Psychiatry, National Clinical Research Center for Mental Disorders, and National Center for Mental Disorders, The Second Xiangya Hospital of Central South University, Changsha, Hunan China; 2 Xiangya School of Medicine, Central South University, Changsha, Hunan China; 3 National Technology Institute on Mental Disorders, Hunan Key Laboratory of Psychiatry and Mental Health, Mental Health Institute of Central South University, Hunan Medical Center for Mental Health, Changsha, Hunan China

**Keywords:** NSSI contagion, Adolescent friendship, Self-esteem, Alexithymia, Self-compassion, Personal distress

## Abstract

**Objective:**

Although empirical evidence of NSSI contagion within adolescent friendships has been documented, the specific mechanisms remain poorly understood. The current study employed a longitudinal design to investigate the influence of NSSI in adolescents’ peer groups on their own NSSI behaviors. Additionally, the study examined the mediating role of self-esteem and the specific conditions under which NSSI contagion occurs.

**Method:**

The study involved 326 adolescents (mean age = 13.5, 59.2% female) nested within 163 friendship dyads. NSSI behavior, self-esteem, self-compassion, alexithymia, and personal distress were assessed at baseline (T1), and NSSI behavior was assessed again after three months (T2). A cross-lagged Actor-Partner Interdependence Model (APIM) was used to estimate the NSSI contagion model. In this model, self-esteem served as a mediator in the contagion of NSSI behaviors, while alexithymia, self-compassion, and personal distress acted as moderating factors of this mediating effect.

**Results:**

This study revealed that friends’ NSSI status at baseline significantly predicted adolescents’ own NSSI status after three months. Self-esteem was found to mediate the NSSI contagion effect exclusively in situations where adolescents exhibited high alexithymia, low self-compassion, and high personal distress.

**Conclusions:**

This research highlights the role of adolescent friendships in NSSI contagion and elucidates the potential mediating role of self-esteem in this contagion. These findings may provide substantial implications for the prevention of the NSSI contagion among adolescents.

## Introduction

Non-suicidal self-injury (NSSI) involves the intentional destruction of body tissue without suicidal intent. This behavior has become a major public health concern, especially among adolescents, who are susceptible to peer influences and social pressures that can exacerbate their vulnerability [1, 2]. Globally, the lifetime prevalence of NSSI averages approximately 28.2%. Among adolescents, the one-year prevalence ranges from 18.1–24.9% [3, 4]. Adolescence is a critical stage where intense peer interaction can influence behavior and emotional health, making adolescents susceptible to social pressures that may contribute to NSSI behaviors [5].

Previous research has suggested that exposure to NSSI behaviors among peers may elicit similar behaviors in adolescents, indicating a potential contagion effect [6]. Further research revealed that adolescents’ exposure to their friends’ self-injurious behaviors significantly elevates their propensity to engage in NSSI, with approximately 38% of adolescents who reported engaging in NSSI citing influence from a peer who exhibited such behaviors [7]. In addition, the frequency of NSSI among friends predicts increased NSSI frequency in adolescents, and social learning theory supports peer influence as a predictor of NSSI behaviors, highlighting the role of peer contagion in its spread [8]. Collectively, the evidence indicates that peer contagion significantly contributes to adolescents’ engagement in NSSI.

To explore the underlying mechanism of NSSI contagion among adolescents, it is crucial to investigate the potential risk factors that mediate the influence of friends’ NSSI behaviors on adolescents’ own actions. Some studies enlightened that low self-esteem maybe a significant predictor of NSSI engagement, as participants who have engaged in NSSI exhibit significantly lower self-esteem, compared to those without a history of NSSI [9, 10]. The relationship between self-esteem and NSSI is often described as bidirectional, with NSSI contributing to further declines in self-esteem through negative self-cognitions, such as guilt and shame, which may arise from societal stigma or other factors [11]. Meanwhile, individuals with low self-esteem tend to experience diminished self-awareness, impairing their ability to employ correct and effective coping mechanisms to alleviate personal distress, and NSSI is often seen as a method to alleviate personal distress [12].

Further, adolescence’s dynamic developmental context necessitates examining potential moderators that influence this mediation. Adolescents with low self-esteem frequently exhibit alexithymia, low self-compassion, and heightened personal distress, which interact with self-esteem to influence NSSI risk [13–15]. First, alexithymia is marked by an inability to identify and verbalize emotions, along with a reduced experience of them [16]. High alexithymia can impede positive self-esteem development due to interpersonal difficulties, and it negatively correlates with self-esteem [17–19]. Second, self-compassion represents a form of self-coping strategy characterized by being friendly, open, forgiving, and non-avoidant [20]. Self-compassionate individuals forgive themselves for mistakes, which fosters positive self-worth evaluations, a key component of self-esteem [21]. Finally, personal distress is an empathy response marked by heightened emotional arousal when confronted with the pain or discomfort of others [22]. Research has identified a positive bidirectional association between distress and NSSI [23]. Thus, adolescents with higher distress may empathically feel their friends’ pain and use NSSI as a coping mechanism, increasing NSSI contagion risk. These factors are closely associated with self-esteem and may serve as moderators that amplify or attenuate the mediation effect of self-esteem on NSSI contagion.

### Objective

Given that the mechanisms underlying NSSI’s contagion remain poorly understood, the study aims to address this gap by exploring the dynamics between adolescents’ NSSI behaviors and their friendships. Specifically, we hypothesize that NSSI exhibits significant actor effects and partner effects in the initial model, and low self-esteem acts as a negative direct mediator in this relationship, while high levels of alexithymia and personal distress, and low levels of self-compassion serve as indirect factors that amplify the aforementioned mediating mechanism.

## Method

### Participants and procedures

The current study was approved by the ethical board of the Second Xiangya Hospital of Central South University. All participants and their parents signed informed consent. The survey was conducted in a junior high school located in Yuxi, China, in September 2022 (Time 1), and December 2022 (Time 2), respectively. Given the less academically busy schedule during which the study took place, adolescents spent a significant portion of their time socializing with friends at school, which made the impact of peer influence especially prominent.

All students (*n* = 1358) in the school were invited to participate in the study using a cluster sampling approach. At Time 1 (T1), 1074 students participated in the study after obtaining parental informed consent. Participants completed peer nominations and a series of psychometric assessments, including the Rosenberg Self-Esteem Questionnaire (RSQ), the Toronto Alexithymia Scale (TAS-20), the Self-Compassion Scale Short Form (SCS-SF), and the Interpersonal Reactivity Index (IRI). Before completing the Ottawa Self-Injury Inventory (OSI), participants were asked about their engagement in NSSI behaviors; those without a history of NSSI were not required to complete this inventory. Of these, 774 participants completed the follow-up assessment at Time 2 (T2), during which NSSI behaviors were reassessed, following the same procedure as the OSI. Participants completed the questionnaire assessment via Wenjuanxing (https://www.wjx.cn/). Participants were given a website link to complete the online survey. Necessary entries need to be completed before submission.

Notably, we employed a cross-lagged design and the Actor-Partner Interdependence Model (APIM) to explore causal relationships. A cross-lagged design was used to investigate the temporal sequence of variable relationships. Data were collected at two time points, allowing us to assess the directionality of effects by comparing cross-lagged paths [24]. This approach is crucial for inferring causality in clinical research [25]. The APIM was utilized to account for interdependent data within dyads. It allows simultaneous examination of actor effects (individual’s influence on own outcomes) and partner effects (individual’s influence on partner’s outcomes). This model helps capture complex interpersonal dynamics and provides a comprehensive understanding of how characteristics and behaviors interact over time [26].

### Measures

#### Demographics

At T1, participants reported their age, gender, grade, whether they have siblings, and whether they are left-behind children of migrant workers, which refers to those under the age of 18 who are left behind in their original residence while one or both parents migrate to other places for work or other reasons. In China, the urban-rural income gap and the hukou system have led to a large number of rural laborers migrating to cities for work, leaving their children typically cared for their grandparents in rural areas [27].

#### Peer nominations

At T1, participants were asked to write down their top five closest friends in order of intimacy [27]. Dyad selection followed specific criteria: first, dyads were formed when both participants listed each other as their closest friend. Second, if one adolescent ranked another as their second-closest friend and was reciprocally ranked as the first choice by that friend, the dyad was selected. Following this, dyads in which both adolescents ranked each other as their second-closest friend were included, with subsequent dyads chosen in a similar manner based on lower rankings. Only participants who completed the T2 follow-up were included in the dyad pairing. To prevent data redundancy, each participant was represented as a “best friend” only once in the dataset. This procedure ultimately yielded a final sample of 326 adolescents, organized into 163 dyads.

#### Non-suicidal self-injury

First, non-suicidal self-injury (NSSI) behavior over the past three months was assessed with the following question: “In the past three months, how many times have you intentionally hurt yourself without suicidal intent?” If participants indicated no engagement in NSSI, they were assigned a score of 0 and this assessment was skipped. For participants reporting NSSI behavior, a 5-point Likert scale was administered based on nine relevant items from the Ottawa Self-Injury Inventory (OSI) [28], with each point representing a different frequency of NSSI behavior. The total score was calculated by summing the scores of the nine items [29, 30]. These items were selected because they were found to be relatively common among adolescents [31], and this measurement demonstrated good with a Cronbach’s alpha (α) coefficient of 0.966 and 0.975 for the T1 and T2 data, respectively. Notably, this study assumed close friendships would demonstrate transparent communication. However, self-reported NSSI does not equate to friends’ awareness, revealing potential informational gaps within social networks.

#### Self-esteem

The Rosenberg Self-esteem Questionnaire (RSQ) is a self-report assessment comprising ten items to measure an individual’s overall sense of self-worth and self-acceptance [32]. Participants were asked to evaluate statements related to themselves on a 4-point Likert scale (1 = completely agree, 4 = completely disagree), such as: “Do you feel you are a person of worth, at least on an equal basis with others?” and “Do you wish you could have more respect for yourself?“. Higher scores on this scale indicate higher self-esteem. The RSQ has demonstrated good reliability and good construct validity in adolescents [33]. In the current study, the RSQ exhibited good internal consistency with a Cronbach’s α of 0.840.

#### Alexithymia

The Toronto Alexithymia Scale (TAS-20) was used to assess alexithymia [34]. This self-report questionnaire consists of twenty questions that specifically address inherent emotions. Each question is rated on a 5-point Likert scale ranging from 1 (strongly disagree) to 5 (strongly agree). The TAS-20 comprises three subscales, which assess difficulty identifying feelings, difficulty describing feelings, and external-oriented thinking, respectively. A higher total score on the TAS-20 indicates a greater level of alexithymia. The TAS-20 demonstrated a good internal consistency in the current study with a Cronbach’s α of 0.836.

#### Self-compassion

Self-compassion was measured by the Self-compassion Scale Short Form (SCS-SF) [35]. This scale consisted of 12 items assessing six dimensions including self-kindness, self-judgment, common humanity, isolation, mindfulness, and over-identification. Each question is rated on a 5-point Likert scale ranging from 1 (strongly disagree) to 5 (strongly agree). Example items include: “I am often confused about what emotion I am experiencing” and “I find it hard to describe how I feel”. A higher total score indicates a higher level of self-compassion (positive aspects are reverse-coded). In general, this scale exhibits a good internal consistency with a Cronbach’s α of 0.86 [35]. In the present study, Cronbach’s α of this scale was 0.752, representing a good level of internal consistency.

#### Personal distress

The Interpersonal Reactivity Index (IRI) is a measure of empathy with subscales assessing cognitive and affective aspects of empathic responding [36]. The Personal Distress (PD) subscale was of interest for the current study. This scale contains seven items that assess an individual’s tendency to become emotionally over-aroused in response to others’ distress. Participants were asked to rate the extent to which each item described them using a 5-point Likert scale ranging from 0 (does not describe me well) to 4 (does describe me well). Example items include: “Do you often try to understand others’ perspectives before responding to a situation?” or “Do you tend to feel emotionally overwhelmed by violent movies or TV shows?”. The Cronbach’s α for this scale was 0.842 in the present study, indicating a high level of internal consistency within this sample.

### Data analysis

Data analyses were performed in the Statistical Package for the Social Sciences (SPSS) 26.0 and Mplus 8.3. For demographic and clinical characteristics, continuous variables were presented as appropriate for percentage or mean and standard deviation, while categorical variables were reported by frequencies and percentages within each category.

Given that data from each participant was nested within dyads, a cross-lagged Actor-Partner Interdependence Model (APIM) was employed to estimate the influences of the targeted adolescent (actor effect) and friend (partner or socialization effect) on the adolescent’ NSSI while accounting for the interdependence of the data [22, 29, 37]. Next, a standard actor-partner interdependence mediation model was constructed to analyze the mediating effect of self-esteem on the relationship between NSSI behaviors in friends at T1 and NSSI behaviors in adolescents at T2. Finally, we tested three moderation models for the above-mentioned mediation model, each of which specified alexithymia, self-compassion, and personal distress as a moderator, separately.

For the initial NSSI contagion model, NSSI was included as a binary variable in the model, given that the relationship between the initial motivation of adolescents engaging in NSSI and NSSI friends could be examined. For the mediator and moderator models, NSSI was included as a continuous variable (sum score of the 9 NSSI behaviors), which offered a more nuanced perspective into how NSSI friends might impact the severity of self-injury among adolescents. Model fit was assessed using Standardized Root Mean Square Residual (SRMR), root mean square error of approximation (RMSEA) [38], the comparative fit index (CFI), and the Tucker-Lewis index (TLI) [39], with smaller values of SRMR and RMSEA and larger values of CFI and TLI indicating better fit [40, 41].

## Results

### Descriptive statistics and correlations

The analysis included data from a total of 326 students, with a mean age of 13.5 years (standard deviation = 0.96), and the sample comprised 59.2% female participants. For other descriptive characteristics, continuous variables were presented as appropriate for mean and standard deviation or percentage in Table 1.

In the present study, 17.48% (*n* = 57) of the participants reported having committed NSSI behaviors at least once in the past three months before participating in the study. NSSI prevalence at T1 and T2 was 9.81% (*n* = 32) and 14.11% (*n* = 46), respectively. Notably, at T1, NSSI prevalence was significantly higher among female participants (87.5%) as compared to male participants (12.5%; χ^2^ = 11.76, *p* < 0.05).

**Table 1:** Characteristics of the participants at T1 and T2

Characteristics	M(SD)/*n* (%)
**Age**	13.5 (0.96)
**Gender**	
Girl Boy	193 (59.2) 133 (40.8)
**Grade**	
Grade 7 Grade 8 Grade 9	96 (29.4) 140 (42.9) 90 (27.6)
**Any siblings**	
Yes No	40 (12.3) 286 (87.7)
**Left-behind children**	
Yes No	9 (2.8) 317 (97.2)

Bivariate correlations among all continuous variables are presented in Table 2. For adolescents and friends, there was a significant positive correlation between NSSI at T1 and T2, and self-esteem was significantly correlated with self-compassion, alexithymia, and personal distress. In addition, it is worth noting that adolescents’ NSSI behaviors at T1 were negatively correlated with friends’ self-esteem and vice versa.

**Table 2:** Means and standard deviations of and bivariate correlations between all continuous variables

	M	SD	1.	2.	3.	4.	5.	6.	7.	8.	9.	10.	11.	12
1. T1 Adolescent NSSI	1.40	4.54	-											
2. T1 Friend NSSI	1.20	3.83	.206^**^	-										
3. T2 Adolescent NSSI	2.15	5.77	.504^**^	0.126	-									
4. T2 Friend NSSI	1.75	5.34	0.099	.455^**^	0.068	-								
5. Adolescent Self-esteem	26.11	6.03	−.348^**^	−.261^**^	−.319^**^	−.231^**^	-							
6. Friend Self-esteem	26.36	5.57	−.158^*^	−.318^**^	-0.150	−.344^**^	.220^**^	-						
7. Adolescent Self-compassion	39.42	7.73	−.246^**^	−.163^*^	−.241^**^	−.225^**^	.667^**^	0.141	-					
8. Friend Self-compassion	39.31	7.36	−.230^**^	−.270^**^	-0.116	−.292^**^	0.140	.666^**^	0.146	-				
9. Adolescent Alexithymia	57.10	9.89	.270^**^	.192^*^	.272^**^	.246^**^	−.676^**^	−.237^**^	−.597^**^	−.218^**^	-			
10. Friend Alexithymia	57.23	11.54	.189^*^	.345^**^	.188^*^	.281^**^	-0.146	−.694^**^	-0.082	−.600^**^	0.146	-		
11. Adolescent Personal distress	13.90	4.76	.217^**^	.231^**^	.284^**^	.271^**^	−.501^**^	−.213^**^	−.441^**^	−.178^*^	.663^**^	0.147	-	
12. Friend's Personal distress	13.21	4.85	.198^*^	.306^**^	.211^**^	.235^**^	-0.128	−.562^**^	-0.022	−.442^**^	.166^*^	.646^**^	.169^*^	-

*** indicates statistical significance at the *p* < 0.001 level; ** at the *p* < 0.01 level; * at the *p* < 0.05 level

### NSSI contagion model (initial model)

**Fig. 1:**
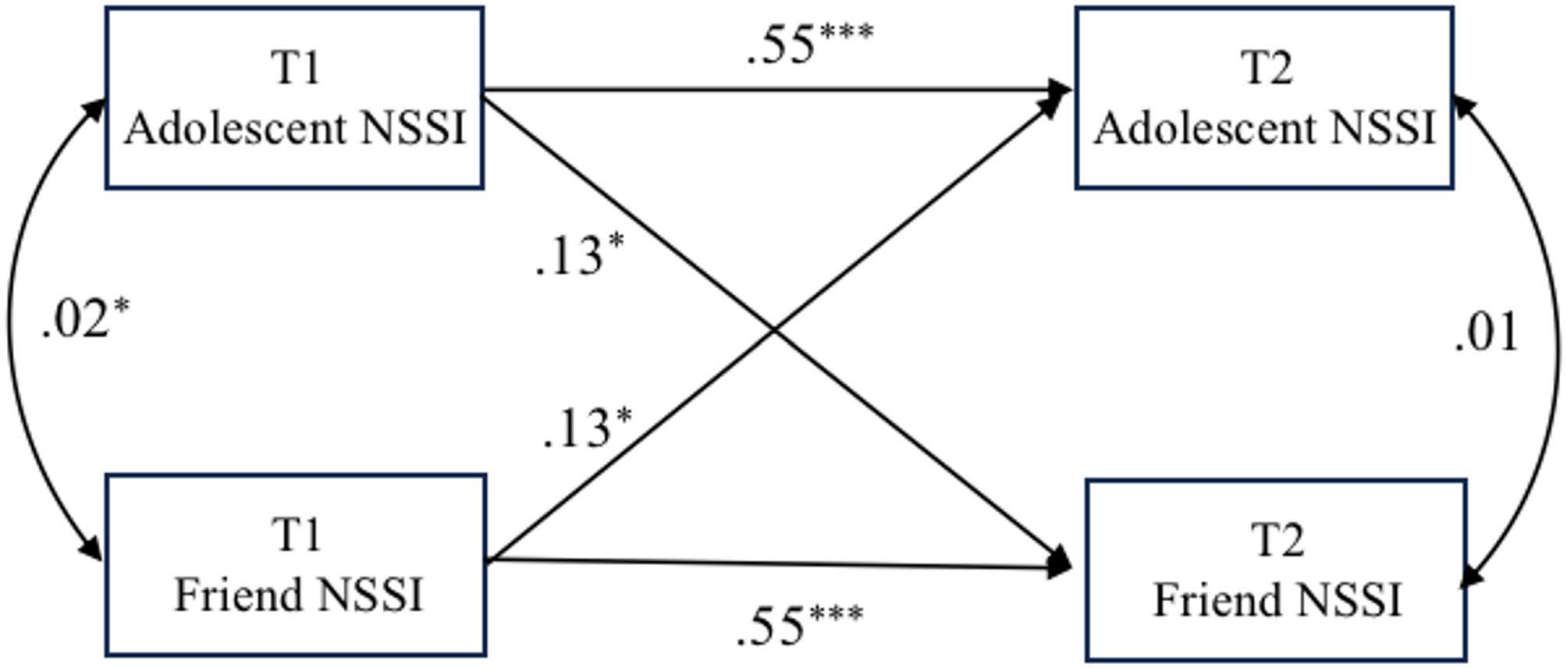
NSSI contagion model

In the assessment of NSSI contagion, the initial (actor-partner interdependence mediation) model was evaluated (Fig. 1). Through analysis, we found that the occurrence of adolescents’ NSSI behaviors at T2 was predicted by their NSSI behaviors at T1 (actor effect). Furthermore, adolescents’ NSSI behaviors at T2 were also predicted by their friends’ NSSI behaviors at T1 (partner effect). The model demonstrated a good level of fit with TLI = 1.04, CFI = 1.00, RMSEA = 0.00, and SRMR = 0.02. The actor effect was significant, indicating the stability of NSSI (β = 0.55, *p* < 0.001). Importantly, the partner effect also was significant, which provides supporting evidence for the NSSI contagion (β = 0.13, *p* < 0.05).

### Self-esteem mediates NSSI contagion

We next tested whether self-esteem acted as a mediator in NSSI contagion. We constructed a standard actor–partner interdependence mediation model to examine the relationships between NSSI behaviors in adolescents and their friends at T1 and T2. In this model, we focused on the path from friends’ NSSI behaviors at T1 to adolescents’ self-esteem, and from adolescents’ self-esteem to their NSSI behaviors at T2.

**Fig. 2:**
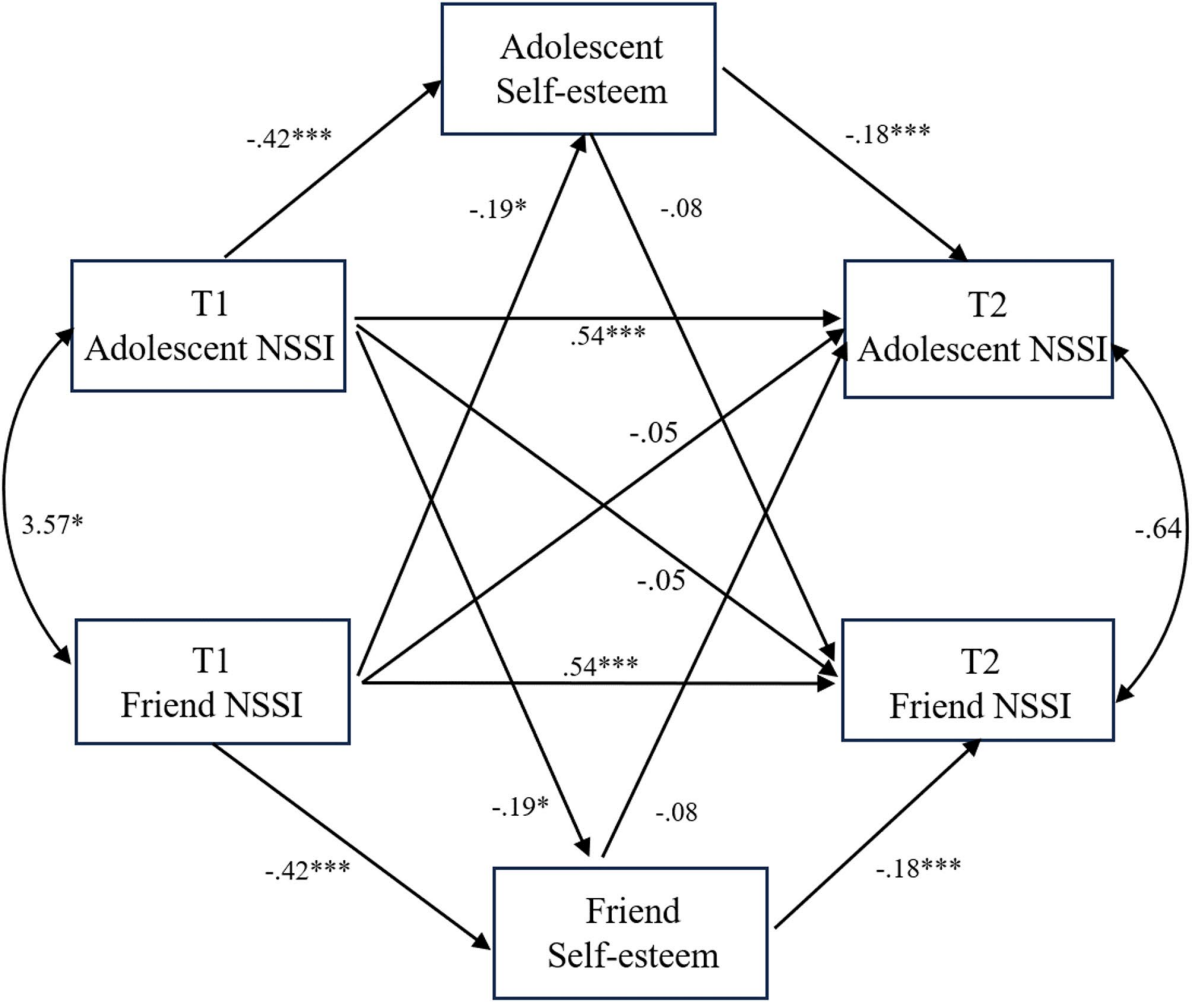
Mediation of NSSI contagion model

The model demonstrated a good fit with TLI = 1.03, CFI = 1.00, RMSEA = 0.00, and SRMR = 0.04. The actor effect was significant, which was consistent with the initial model (β = 0.54, *p* < 0.001). Friends’ NSSI behaviors at T1 were found to significantly predict adolescents’ self-esteem (β = −0.19, *p* < 0.05), and adolescents’ self-esteem significantly predicted their own NSSI behaviors at T2 (β = −0.18, *p* < 0.001). The partner effect was no longer significant and dropped from β = 0.13 (*p* < 0.05) in the initial contagion model (Fig. 2) to β = −0.05 (*p* > 0.05) in the current model with self-esteem.

**Fig. 3:**
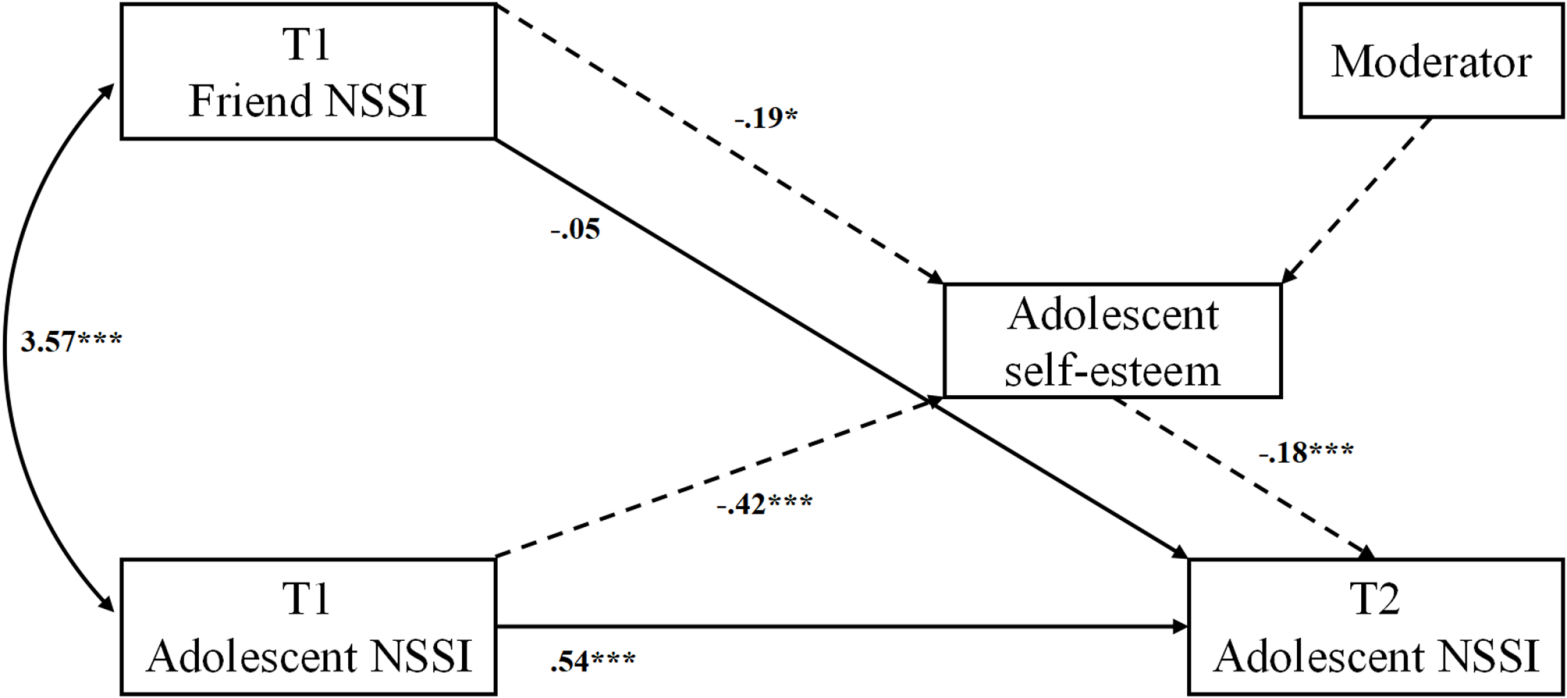
Simplified moderated mediation of NSSI contagion model. (duplicate paths not shown)

To test our moderated mediation hypothesis, two paths were added: one path from the moderator of interest to adolescents’ NSSI behavior at T2 and another path from the interaction variable (product of self-esteem variable and moderator) to adolescents’ T2 NSSI behaviors. The significance of the interaction effect was tested in each model, with the simplified model presented (Fig. 3).

**Fig. 4:**
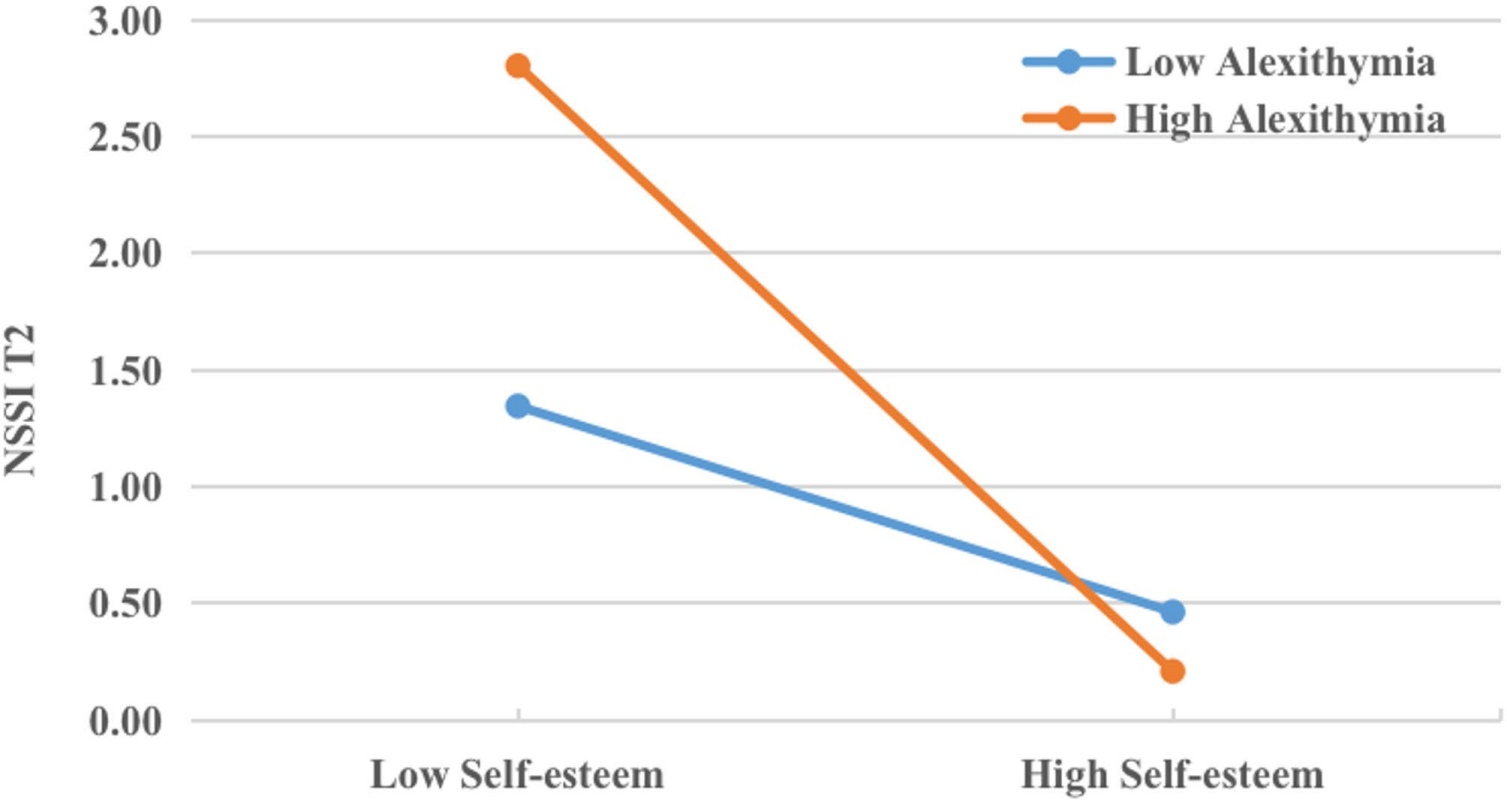
Indirect effect of self-esteem on T2 adolescent NSSI versus the moderator. (Moderated mediation with alexithymia)

Firstly, a simple slopes test was conducted to examine the moderating effect pattern of alexithymia (Fig. 4). Analyses first tested the moderating effect of alexithymia. Conditional indirect effect of self-esteem was calculated at low (− 1 SD) and high (+ 1 SD) levels of adolescents’ alexithymia, and tests of simple slopes were conducted to illustrate the patterns of alexithymia’s moderation effects. The interaction between self-esteem and adolescents’ alexithymia yielded a significant result, with a coefficient of β = −0.428, 90%CI: −0.792 to −0.063. Results revealed that the indirect effect of self-esteem was more pronounced for adolescents with high levels of alexithymia (IE = 0.045; 95%CI: 0.002–0.087) than for those with low levels of alexithymia (IE = 0.015; 95% CI: −0.016 to 0.047), indicating that high alexithymia strengthened the negative effects of self-esteem on NSSI at T2.

**Fig. 5:**
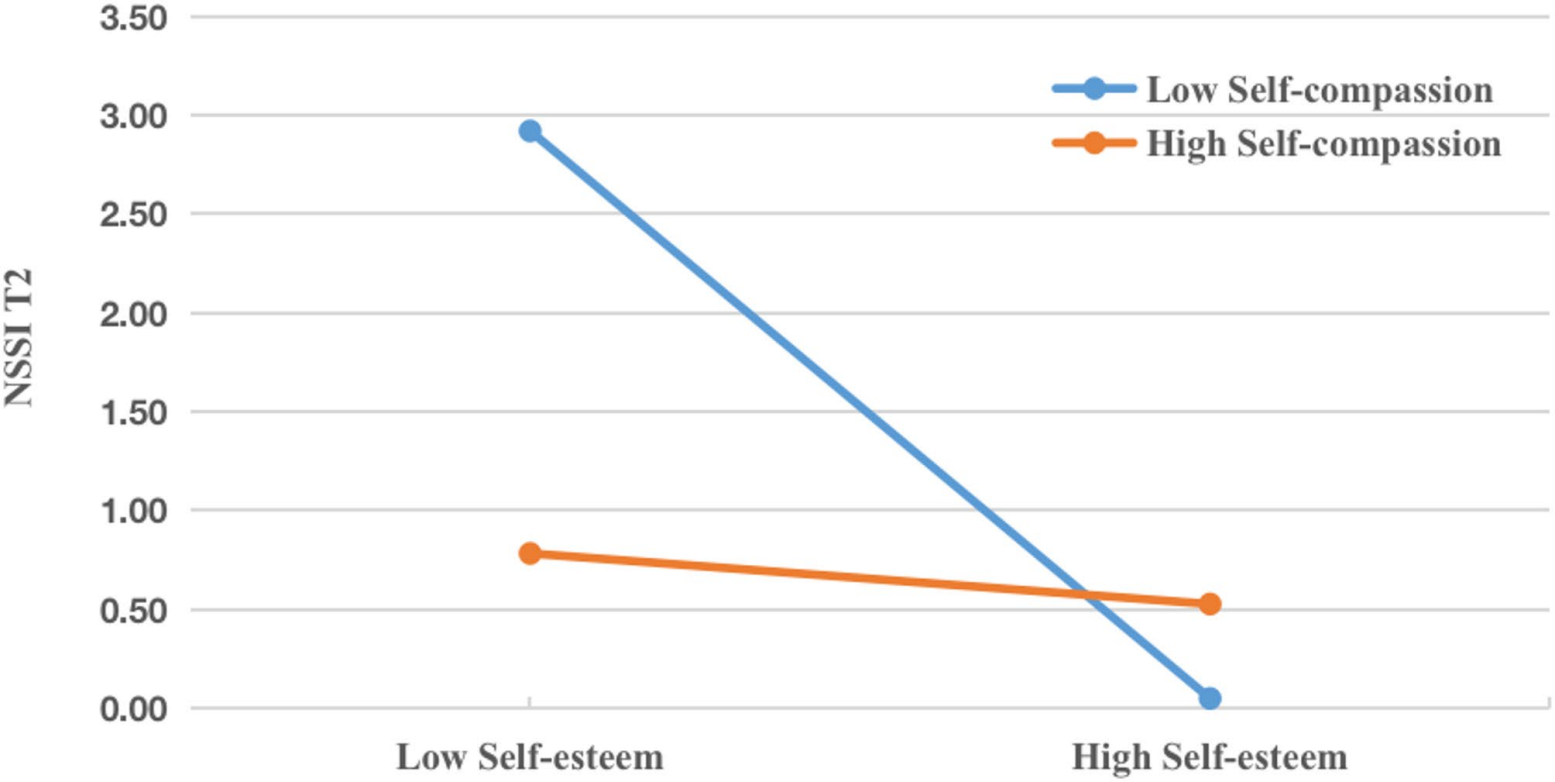
Indirect effect of self-esteem on T2 adolescent NSSI versus the moderator. (Moderated mediation with self-compassion)

Analyses then tested the moderating effect of adolescents’ self-compassion (Fig. 5). These analyses were closely parallel with those conducted to test the moderating effect of alexithymia. The interaction between self-esteem and adolescents’ self-compassion was significant (β = 0.654, *p* < 0.05). Specially, the conditional indirect effect of self-esteem was significant at low levels of self-compassion (IE = 0.049, 95%CI: 0.004–0.095). Namely, a low level of self-compassion strengthened the mediational effects of self-esteem on NSSI at T2. Although the conditional indirect effect of self-esteem at high levels of self-compassion was not significant (IE = 0.004, 95%CI: −0.025 to 0.033), high self-compassion itself served as a protective factor that diminished the risk of NSSI at time two regardless of self-esteem.

**Fig. 6:**
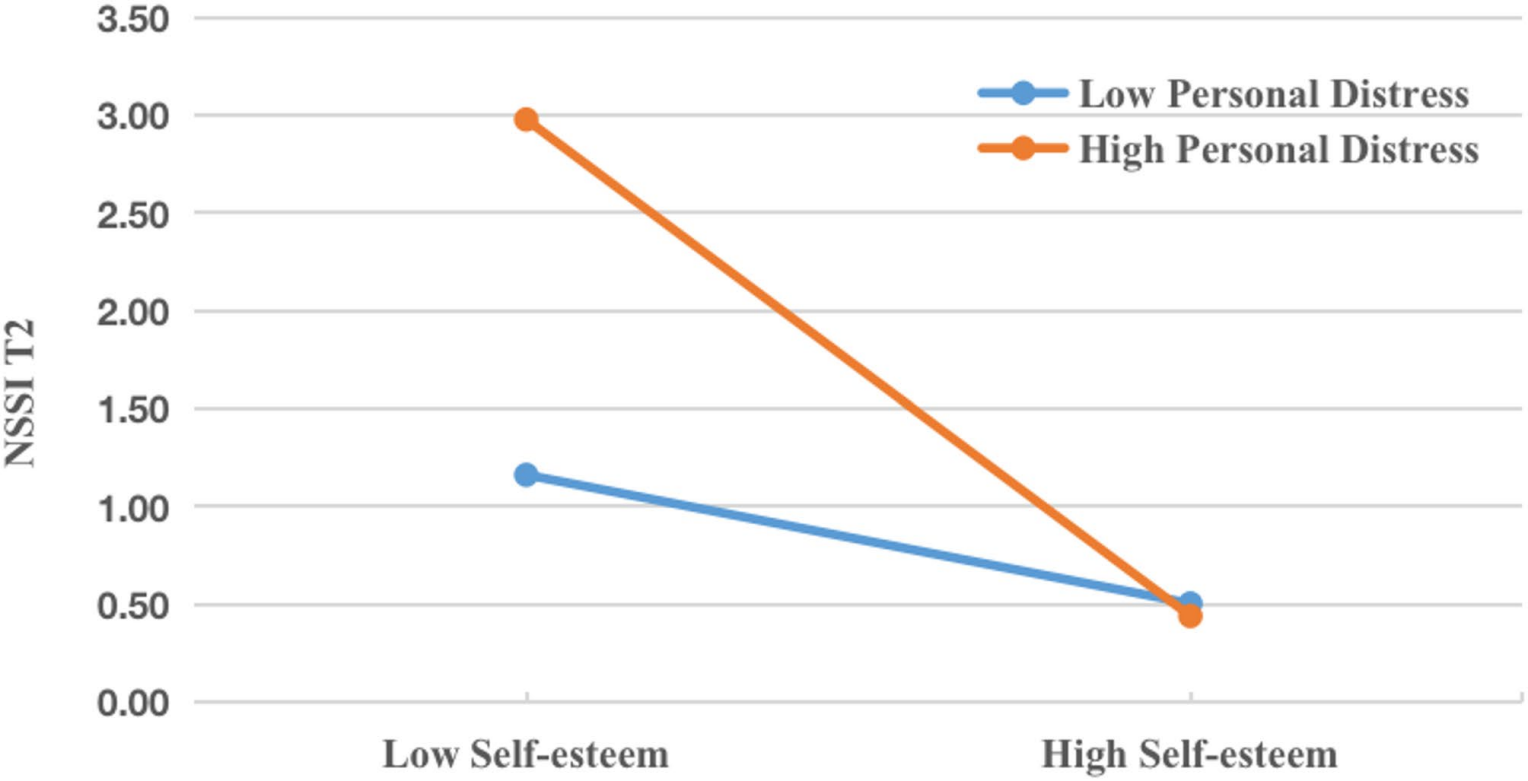
Indirect effect of self-esteem on T2 adolescent NSSI versus the moderator. (Moderated mediation with personal distress)

Lastly, we tested the moderating effect of personal distress (Fig. 6). The interaction between self-esteem and adolescents’ personal distress produced a significant result, with a coefficient of β = −0.470, 90% CI: −0.867 to −0.073. Analyses indicated that the conditional indirect effect of self-esteem was significant at high levels of personal distress (IE = 0.044, 95% CI: 0.002–0.085). While at low levels of personal distress, the conditional indirect effect of self-esteem was not significant (IE = 0.011, 95% CI: −0.017 to 0.040). These results suggest that adolescents may be more susceptible to NSSI contagion via self-esteem when their personal distress is higher than others.

## Discussion

In this study, we preliminarily constructed an NSSI contagion model using a longitudinal cross-lagged design with the Actor-Partner Interdependence Model (APIM), where self-esteem served as a mediator, and alexithymia, self-compassion, and personal distress acted as moderators. This contagion model may provide innovative insights into pathways for understanding NSSI contagion among adolescents.

First, this study found evidence for the socialization of NSSI within adolescent friendship dyads. Namely, friends’ NSSI status may be a potential predictor for adolescents’ own NSSI status. Based on past empirical evidence, NSSI contagion is commonly observed in several settings, including hospitals, communities, and online platforms [6]. This phenomenon may even have a global presence among adolescents, highlighting the need for increased public and professional awareness and attention. While previous research provided initial insights into NSSI contagion, our study consolidates the existing evidence on NSSI contagion to a certain extent.

Second, the results of the current study supported that the self-esteem might act as a mediation role in the contagion effects, as it manifested that NSSI behaviors in friends would negatively affect adolescents’ self-esteem but could potentially contribute to adolescents’ engagement in self-injurious behaviors. This result aligns with previous findings, which suggest that having a greater number of acquaintances engaged in NSSI is correlated with lower levels of self-esteem [42]. The possible underlying reasons for this connection are as follows. On the one hand, the relationship aligns with the experiential avoidance model, where NSSI temporarily alleviates aversive states linked to poor self-worth. For example, adolescents with lower self-esteem are more likely to engage in NSSI to regulate intense shame or self-disgust [11, 43]. On the other hand, the social comparison theory suggests that during interactions with friends, adolescents unconsciously compare their thoughts, emotions, and behaviors with those of their friends, and exhibit a tendency towards assimilation in the process [44]. Given that individuals with NSSI behaviors report lower self-esteem, it is possible for adolescents to inadvertently acquire lower self-esteem from their friends who engage in NSSI behaviors through the assimilation process described by social comparison theory [45, 46]. Subsequently, when adolescents encounter difficulties in self-acceptance, emotion regulation, or interpersonal relationships, they are likely to use NSSI as a strategy for self-relief.

Besides, preliminary evidence indicates that heightened alexithymia and personal distress, alongside reduced self-compassion, could interact with self-esteem to influence pathways associated with NSSI contagion. The effect of self-esteem on NSSI behaviors is supported by a theoretical framework suggesting that negative self-beliefs can influence an individual’s willingness to tolerate pain. Over time, heightened intolerance of pain may trigger NSSI as a coping mechanism [47]. Adolescents with alexithymia may misinterpret peers’ NSSI behaviors as normative coping strategies, potentially adopting similar actions to manage unresolved distress. This emotional misunderstanding could simultaneously erode self-esteem, as persistent challenges in identifying and articulating emotions may intensify negative self-perceptions, thereby reinforcing vulnerability to behavioral contagion [43, 48]. Additionally, individuals with low self-compassion are particularly at risk, as their self-critical tendencies might cause them to perceive NSSI as a way to seek peer validation or to punish themselves for perceived shortcomings. This may contribute to a cycle of diminished self-esteem and heightened reliance on maladaptive social reinforcement [6, 49]. Moreover, personal distress may prompt adolescents to imitate their peers’ NSSI as a misdirected attempt to regulate their own emotional arousal, which could negatively impact self-esteem by linking self-injury to relational survival, where maintaining social bonds may seem to require adopting peers’ harmful behaviors [29, 50]. Collectively, these specific conditions may contribute to a vulnerable environment where NSSI ideas become more contagious.

Notably, some socioeconomic factors may significantly influence adolescent vulnerability to NSSI as well. For example, adolescents experiencing low socioeconomic status or prolonged parental absence face compounded risks for NSSI. Studies show that lower parental income and education levels correlate with a 1.08–1.19× higher likelihood of self-harm, particularly among girls, even after adjusting for confounders like psychiatric diagnoses [51, 52]. Chronic parental absence (whether physical or emotional) may exacerbate psychosocial risks by undermining core self-worth development. Specifically, paternal absence disproportionately impacts males’ self-esteem trajectories, whereas emotionally distant parenting practices may impair self-assessment capacities and adaptive coping mechanisms [53, 54]. This erosion of self-esteem is critical, as low self-worth amplifies frustration tolerance deficits and hostility, both linked to NSSI. Further, socioeconomic deprivation limits access to mental health resources, compounding the effects of familial instability [55].

At last, our findings should be interpreted in light of several limitations. Firstly, while our study focused on NSSI contagion in constructed friendship networks, a significant amount of data had to be excluded, potentially leading to an incomplete network representation. Besides, we have only elucidated one contagion model, and more alternative models remain to be further explored. Secondly, our model examined self-esteem as a mediator of NSSI contagion, with self-compassion, alexithymia, and personal distress as moderators, but did not explore other potential mediating factors due to the complexity of adolescence. Thirdly, our data was collected from junior high school students in a small county town in China, limiting the generalizability of our model beyond this context, although it is relevant for tailored intervention strategies in developing countries. Then, a key methodological limitation is the insufficient elaboration of self-esteem as a mediator, as specific peer relationship dimensions influencing self-esteem were not dissected. This stems from our composite assessment of peer relationship quality, which may conflate distinct pathways through which peer experiences shape self-esteem and downstream NSSI risk. Future research should prioritize granular assessments of peer dynamics to clarify these mechanisms with precision. Lastly, our study was limited to two time points, restricting our ability to fully validate and refine the model’s mechanisms. Specifically, the temporal precedence of the mediator relative to the outcome cannot be robustly established when both are measured concurrently at Time 2. This design precludes the examination of dynamic, bidirectional processes and limits the capacity to model non-linear trajectories in socialization mechanisms. Furthermore, the compressed timeframe may obscure critical developmental windows during which peer influences on NSSI contagion are most potent, potentially conflating proximal and distal socialization effects.

## Conclusion

In this study, we preliminarily established an NSSI transmission model and found that self-esteem may play a potential mediating role in the process of NSSI contagion. Furthermore, we observed that the mediating effect of self-esteem is more pronounced in individuals with higher levels of alexithymia, lower self-compassion, and greater personal distress. These findings highlight the potential benefits of tailored interventions for at-risk groups and provide further insights into the understanding and prevention of NSSI contagion.

## Data Availability

No datasets were generated or analysed during the current study.
